# Heliaphen, an Outdoor High-Throughput Phenotyping Platform for Genetic Studies and Crop Modeling

**DOI:** 10.3389/fpls.2018.01908

**Published:** 2019-01-16

**Authors:** Florie Gosseau, Nicolas Blanchet, Didier Varès, Philippe Burger, Didier Campergue, Céline Colombet, Louise Gody, Jean-François Liévin, Brigitte Mangin, Gilles Tison, Patrick Vincourt, Pierre Casadebaig, Nicolas Langlade

**Affiliations:** ^1^LIPM, INRA, CNRS, Université de Toulouse, Castanet-Tolosan, France; ^2^AGIR, INRA, Université de Toulouse, Castanet-Tolosan, France; ^3^UE Auzeville, INRA, Castanet-Tolosan, France

**Keywords:** plant phenotyping, robotics, water stress, genomics, crop modeling

## Abstract

Heliaphen is an outdoor platform designed for high-throughput phenotyping. It allows the automated management of drought scenarios and monitoring of plants throughout their lifecycles. A robot moving between plants growing in 15-L pots monitors the plant water status and phenotypes the leaf or whole-plant morphology. From these measurements, we can compute more complex traits, such as leaf expansion (LE) or transpiration rate (TR) in response to water deficit. Here, we illustrate the capabilities of the platform with two practical cases in sunflower (*Helianthus annuus*): a genetic and genomic study of the response of yield-related traits to drought, and a modeling study using measured parameters as inputs for a crop simulation. For the genetic study, classical measurements of thousand-kernel weight (TKW) were performed on a biparental population under automatically managed drought stress and control conditions. These data were used for an association study, which identified five genetic markers of the TKW drought response. A complementary transcriptomic analysis identified candidate genes associated with these markers that were differentially expressed in the parental backgrounds in drought conditions. For the simulation study, we used a crop simulation model to predict the impact on crop yield of two traits measured on the platform (LE and TR) for a large number of environments. We conducted simulations in 42 contrasting locations across Europe using 21 years of climate data. We defined the pattern of abiotic stresses occurring at the continental scale and identified ideotypes (i.e., genotypes with specific trait values) that are more adapted to specific environment types. This study exemplifies how phenotyping platforms can assist the identification of the genetic architecture controlling complex response traits and facilitate the estimation of ecophysiological model parameters to define ideotypes adapted to different environmental conditions.

## Introduction

Fertilizers, irrigation, and pesticides used to mitigate the effects of climatic hazards had a large, positive impact on crop yields between 1960 and 2000 (Tilman et al., 2002; Foley et al., 2005). The current need to reduce inputs in agricultural systems while coping with the climatic uncertainty caused by climate change means that farming conditions have become more variable than they were in the late 20th century. Strategies to develop highly plastic crop genotypes or predict the best combinations of genotypes and agro-management practices for local conditions are therefore key targets. Regardless of the magnitude of changes required in farming systems (Duru et al., 2015), achieving these goals will require the characterization and elucidation of how plants adapt to their environment.

Climate change scenarios indicate that summer precipitation will substantially decrease in Southern and Central Europe, and to a smaller degree in Northern Europe, in the future; however, during spring and autumn, the precipitation change should be marginal (Moriondo et al., 2010). Drought episodes will therefore continue to occur, probably with increasing variability from year to year. In an agricultural context, a drought-tolerant plant is one that maintains growth and production during gradual and moderate soil water deficits, ideally without exhibiting protection mechanisms (Tardieu, 2011). Water deficit affects a large spectrum of plant functions, such as transpiration, photosynthesis, leaf and root growth, and reproductive development (Chaves et al., 2003), by impacting the underlying physiological processes (e.g., cell division, primary and secondary metabolism) (Tardieu et al., 2018). Drought tolerance is the result of integrated processes taking place at different timescales to produce a long-term impact on leaf growth and transpiration (Tardieu et al., 2018).

In Europe, sunflower (*Helianthus annuus*) is largely a rainfed crop, and water deficit is frequently the main factor limiting its yields (Blanchet et al., 1981; Debaeke et al., 2017). Sunflower drought responses have long been studied at the physiological level, and more recently at the molecular level. Different researchers have characterized the impact of water deficit on leaf development, transpiration, photosynthesis, and biomass allocation processes (Adiredjo et al., 2014a,b; Andrianasolo et al., 2016a,b; Pereyra-Irujo et al., 2008; Velázquez et al., 2017), revealing differences in these traits between genotypes with lower and higher water use efficiencies. The molecular pathways underpinning these processes have begun to be described at the transcriptomic level, elucidating the role of osmotic potential maintenance under both controlled conditions and in the field (Rengel et al., 2012), the importance of reactive oxygen species (ROS; Ramu et al., 2016), and highlighting the role of phytohormone signaling pathways, including abscisic acid (Rengel et al., 2012; Sarazin et al., 2017), ethylene (Manavella et al., 2006), and jasmonate signaling (Marchand, 2014; Andrade et al., 2017). In a holistic approach, Marchand (2014) inferred a gene regulation network in the drought response based on hormonal signaling pathways, revealing the role of drought tolerance in the evolution of wild sunflowers and in modern breeding. Also, Clauw et al. (2016); Rymaszewski et al. (2017), and Tardieu et al. (2014) combined genetical genomics approaches (mostly transcriptomics) to eco-physiological description of drought stress response in *Arabidopsis* to understand their genetic and molecular control. The above ecophysiological and molecular insights are based on simple drought stresses, mostly during early vegetative development; therefore, it is vital to continue to develop our knowledge using more complex and realistic scenarios, taking into account the dynamic and organ-level impacts of drought stresses and plant responses.

The different soils and climates of various cropping environments in combination with plant genotypes and the management conditions used can generate highly diverse water deficit scenarios (Chenu et al., 2013). A given trait can therefore have positive, null, or even negative impacts on crop performance in different drought scenarios (Casadebaig et al., 2016b; Millet et al., 2016). Because plant traits of interest are context dependent, we need tools to measure and integrate the multiple overlapping mechanisms involved in plant responses to water deficit, among which plant growth and transpiration are the primary targets (Tardieu et al., 2018). Phenotyping platforms and crop mathematical models can be used as complementary tools to address this objective.

Driven by technological development, phenotyping platforms are tools created to efficiently measure plant traits while controlling cultivation conditions. Greenhouse-based platforms allow a very fine control of the environment (light, water, and nutrients; Granier et al., 2005; Pieruschka and Poorter, 2012; Cobb et al., 2013), and have been successfully used to conduct association genetic studies (Cabrera-Bosquet et al., 2012) and derive plant trait ranges that can be used as inputs into crop models (Steduto et al., 2009; Lenz-Wiedemann et al., 2010). The design factors that are important for achieving high-throughput phenotyping (small plots and plants) and a careful control of environmental conditions (closed facility, artificial lighting) mean that cultivation conditions can be quite different from conditions in the field however, which may cause difficulties when generalizing plant responses to agricultural conditions (Mittler, 2006).

Computer-based plant modeling approaches have recently emerged as a method to complement and improve the resource-limited experimental exploration of the adaptation landscape (Chapman et al., 2003; Hammer et al., 2006; Messina et al., 2006, 2011). These software models are based on mathematical equations that represent the biological processes linked to plant growth and development as a function of time, environment (climate, soil, and management), and genotype-dependent parameters. The genotype-dependent parameters used are expected to be more heritable than complex traits, which are generally more influenced by environmental variation and genotype–environment interaction (Heslot et al., 2014). Accordingly, simulations can be used as a tool to predict the trait × trait and trait × environment interactions and facilitate the assessment of the importance of a particular trait when scaling up from the individual plant to a plant population (one field) or to a crop population over several fields in a cropping region. Simulations have been successfully used to assess resource availability over time and reveal stress scenarios at a continental scale (Chenu et al., 2013), to search for traits adapted for specific environment types (Chapman et al., 2002; Casadebaig et al., 2016a), and to infer trait values beyond field experiments and available genetic diversity (Martre et al., 2015; Casadebaig et al., 2016b).

In this context, we aimed to characterize various sunflower genotypes using a phenotyping platform measuring genotype-dependent traits, thus enabling the simulation of genotype performance in diverse abiotic stress scenarios. Ultimately, linking phenotyping, crop modeling, and simulation would allow us to design a decision support system to better select genotypes most suited to a particular cropping environment, accounting for climatic uncertainty.

To achieve this goal, we developed the Heliaphen phenotyping platform to measure key phenotypic traits involved in the plant response to water deficit across large panels of genotypes. In order to study traits related to crop performance (e.g., seed number and mass, seed oil content), the plants are grown in outdoor conditions with automatically managed water deficit scenarios and plant trait measurements. The use of daily measurements of leaf and plant growth rates, plant transpiration rates (TRs), and water deficit levels allowed the estimation of the phenotypic response to water deficit (reaction norms). We also estimated the parameters of the response curves of leaf expansion (LE) and plant transpiration to varying water deficits using non-linear regression, and used these genotype-dependent parameters in a crop simulation model (SUNFLO; Casadebaig et al., 2011) to simulate the performance of the phenotyped genotypes in non-observed environments.

**FIGURE 1:**
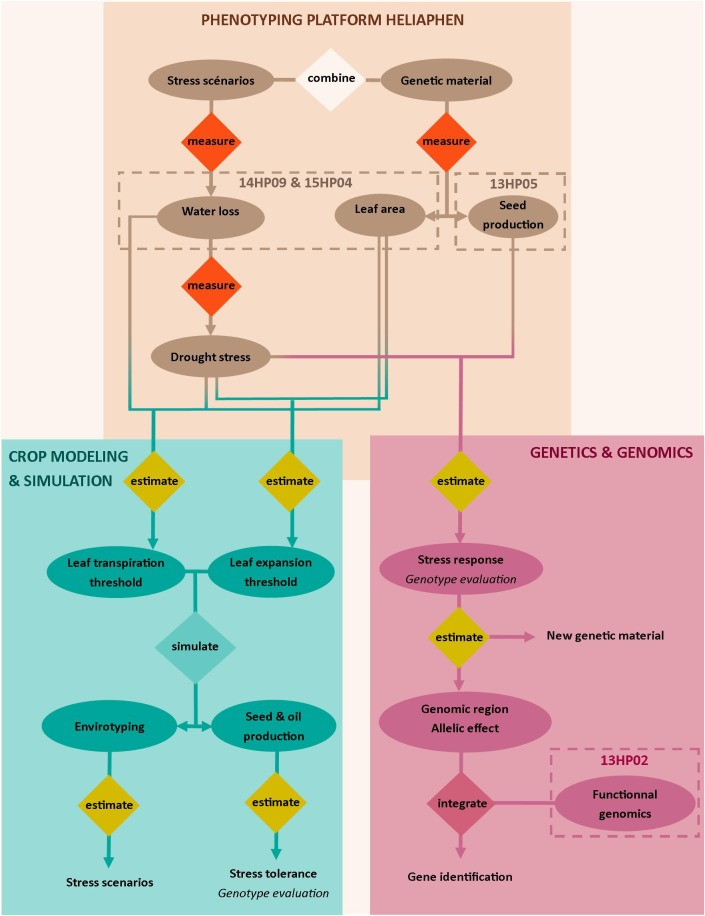
Overview of the approaches targeted with the Heliaphen phenotyping platform. This figure illustrates how we used the phenotyping platform to (1) grow diverse genetic material under designed stress scenarios and measure key ecophysiological traits and plant water status (light beige box), (2) conduct genetic and genomic studies on measured phenotypes (pink box) to identify gene functions, and (3) estimate crop simulation parameters based on the measured phenotypes to be used in simulation studies. The simulation study allows the characterization of abiotic stress responses at the crop level (envirotyping) and the evaluation of genotype value (performance and stress tolerance) in diverse cropping conditions (blue box). Water stress is indicated by the fraction of transpirable soil water (FTSW). Codename of experiments are specified in the dotted boxes.

Here, we validated our ability to use the Heliaphen platform to manage distinct drought scenarios impacting plant leaf area, biomass, and yield-related traits (green box, Figure 1). Two distinct approaches were then undertaken as case studies to demonstrate the capabilities of the platform. The first approach validated its use for genetic and genomic studies; the platform data was used for an association study and transcriptomics analysis to identify the genetic basis of the response of the yield-related traits to water deficit (blue box, Figure 1). The second study illustrated how the traits measured using the phenotyping platform could be used in a crop simulation model to analyze how they impact yields in diverse and non-observed cropping conditions (pink box, Figure 1).

## Materials and Methods

### Heliaphen Platform

**FIGURE 2:**
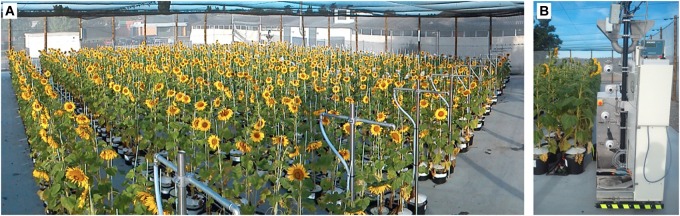
General view of the Heliaphen platform. **(A)** The Heliaphen platform comprises an area of 650 m^2^, containing 1,300 plants spread over 13 blocks consisting of two rows of 50 pots each. **(B)** The Heliaphen robot is able to pick up pots, weigh and water them, and phenotype individual plants.

Heliaphen is a 650 m^2^ outdoor phenotyping platform in which a robot performs measurements and irrigates plants grown in pots. Heliaphen can hold up to 1,300 plants spread over 13 blocks consisting of two rows of 50 15-L pots each (Figure 2A). This outdoor platform is surrounded by nets to deflect wind and prevent bird entry. In order to control the water balance and manage water deficit scenarios, the pots are covered with a cone-shaped cap to prevent rainwater from entering the pot. Environmental conditions are recorded and logged, including temperature, wind, precipitation, and evaporative demand.

The robot (Figure 2B) is based on an electrical propulsion system. Its batteries allow about 48 h of continuous function before requiring recharging for about 4 h. It is equipped with a 60-L water tank used to manage irrigation. The robot’s navigation within the platform and its management of water and electricity use are fully autonomous. The robot is equipped with a clamp to enable it to pick up pots, a digital scale (Midrics 1, Sartorius Weighting Technology GmbH, Gettingen, Germany), and four digital cameras (Prosilica GC650 with Sony ICX424 sensor and 659 × 493 resolution). Weighing, irrigating, and image acquisition for each plant lasts between 30 and 90 s, depending on the amount of water required. The weight of the pots is recorded before and after irrigation, and the plants are photographed using the cameras.

The robot manages drought scenarios at the plant level, i.e., for each pot. Before the beginning of the stress period (early vegetative stage), pots are irrigated to saturation, and after the excess water has drained, the pots are weighed to estimate the pot mass at full soil water capacity. For each plant, the planned water deficit level, expressed as a fraction of transpirable soil water (FTSW; Sinclair et al., 2005), is converted to a target pot weight and logged into the Heliaphen software. For each interaction of the robot with the plants, the robot navigates the plant stand, picks up a pot using its clamp and weighs it, then irrigates the plant according to the defined water deficit scenario, which in practice means adding sufficient water to correct the difference between the actual and target pot weights. Both control and stressed plants are processed one to four times per day by the robot to estimate plant transpiration according to the experiment size and required phenotyping intensity. In addition, an independent drip irrigation system is used to support plant growth before robot-dependent, controlled irrigation management. This allows different irrigation and fertilization regimes using a liquid nutrient supply managed using a Dosatron system (Dosatron International, Tresses, France).

In addition to the high-throughput automated measurements, manual measurements are currently required for the assessment of other plant traits in the Heliaphen, such as individual leaf area and plant height. The platform was developed using sunflower as a model species; however, maize (*Zea mays*), soybean (*Glycine max*), and tomato (*Solanum lycopersicum*) have been successfully tested on it.

### Plant Material, Experiments, and Multi-Environment Trials

Various panels of sunflower plant materials were used for different experiments in this study (summarized in Table 1), and are briefly described below, with the full description available in the supplementary material provided by Badouin et al. (2017). Each trial was assigned an identifier consisting of two numbers denoting the year, followed by two letters encoding the location (HP for experiments on the Heliaphen platform, or EX or RV for field experiments), and two final numbers denoting the trial number.

**Table 1:** Plant material and experiments used in this study.

		Genetic	Genotype
Trial name	Panel name	characteristics	number	Replicates	Phenotyped traits	Environment
13HP05	INEDI RIL	Backcrossed RILs	88	4	TKW, SFTSW	Heliaphen
16HP07	NK KONDI	Hybrid	1	6	Plant weight, seed weight, TKW, SFTSW	Heliaphen
14HP10	SUNRISE hybrid panel	Hybrids	429	1	Seed weight	Heliaphen
14RV01, 13EX01, 13EX02, 13EX03, 13EX04, 13EX05, 14EX04, 14EX05, 15EX05, 15EX07	SUNRISE hybrid panel	Hybrids	426 to 482	1 to 2	Seed weight	Field
14HP09, 15HP04	Commercial varieties	Hybrids	16	6	Transpiration rate, leaf expansion rate	Heliaphen
13HP02	SUNRISE parent and hybrid panel	Hybrids and parental lines	2 out of 24	3	Transcriptome	Heliaphen
S0531, S0631, S0634, S0731, S0831, S1031	Post-inscription commercial varieties	Hybrids	9 to 21		Transpiration rate, leaf expansion rate	Greenhouse


#### General Validation of the Phenotyping Platform

The results of experiments performed on the Heliaphen platform were validated by comparing performance-related traits between plants grown in pots and in the field (comparison of trials 14HP10 and 14RV01). These two experiments were conducted using the same genetic panel (490 hybrids; Table 1) sown at the same time (April 30 and May 5, 2014) in the same geographical location (the trials were 4.11 km apart). Other field experiments (13EX01 to 15EX07) were conducted on the same hybrid panel at different locations and sown on different dates. The seed weights of plants grown in the 14HP10 trial were estimated by sampling individual plants from each plot, whereas in the other trials, it was computed as the ratio of harvested seed weight to the number of plants per plot.

The platform capacity to generate distinct water deficit levels and measure their direct impacts on performance-related traits was determined in trial 16HP07. This trial involved one genotype, the commercial hybrid NK KONDI, subjected to different drought scenarios. Seven different levels of water stress were applied during the reproductive stage (flowering to harvest), with six replicates for each stress. The target relative stress levels were 0.2, 0.3, 0.4, 0.6, 0.75, 0.9, and 1, expressed in FTSW, where 1 corresponds to the maximum amount of water available to plants (meaning the plant is not water stressed).

#### Trials Used for Genetic and Genomic Studies

The genetic study was performed using the 13HP05 Heliaphen trial. The tested panel was a recombinant inbred line (RIL) population derived from the cross between lines XRQ/B (SF193) and PSC8/R (SF326). To overcome disease-sensitivity issues caused by the duration of the experiment and the sensitivity of the PSC8 genotype to *Verticillium* and *Alternaria* fungi, and to avoid branched plants, 84 RILs carrying the Rf1 fertility restoration allele were backcrossed to the non-branched male-sterile parent XRQ/A (SF193). Two drought scenarios were conducted; the plants either received normal irrigation or a constant water deficit level of FTSW = 0.4 from flowering to harvest. Four replicates were used for each condition.

The transcriptomic study was performed on plants grown in the 13HP02 Heliaphen trial. This trial was composed of 24 genotypes; four female lines, four male lines, and their 16 hybrids. The panel included the hybrid SF193 × SF326 and its parental lines, the results of which are presented below. Two drought scenarios were applied during the vegetative stage, a control (irrigated) and a progressive water deficit (non-irrigated) scenario, with three replicates of each genotype for each condition. The treatments started 35 days after germination, when irrigation was stopped for the stress treatment plants. To ensure a comparable level of water stress between individual plants for the transcriptomic analysis, pairs of stressed and control plants were harvested when the FTSW of the stressed plant reached 0.1.

#### Trials Used for Crop Modeling and Simulation

The comparisons of the response traits of plants grown in the Heliaphen platform and the greenhouse were performed using trials 14HP09 and 15HP04 for the Heliaphen platform, and greenhouse trials S0531, S0631, S0634, S0731, S0831, and S1031. In these previous trials (Casadebaig et al., 2008), a total of 82 commercial sunflower hybrids were phenotyped to determine their LE and TRs in response to water deficit. Only three control genotypes were common among all trials; therefore, the comparison was performed using the mean and the variance of the traits across the trialed populations. To estimate the response traits for any particular genotype, the leaf area expansion rate, TR, and water deficit levels were measured for 12 plants growing under two drought treatments (control – irrigated – and a progressive water deficit – non-irrigated), defined during the vegetative stage. Six replicates were performed for each scenario. The response traits were determined as the regression of the phenotype (expansion and transpiration) response (ratio of each stressed plant to the average of the control plants) to the water deficit level, described in full by Casadebaig et al. (2008).

### QTL Detection

To demonstrate the capacity of identifying genetic markers associated with the water stress response of a yield component, a genetic study was performed using data generated in the 13HP05 trial. The observation of the thousand-kernel weights (TKWs) achieved using the Heliaphen platform, together with the available genotyping data for the population used in this trial (from Badouin et al., 2017), enabled QTL detection using a model based association method (Yu et al., 2006). The population of RILs used in the 13HP05 trial was grown in control conditions or submitted to drought stress (FTSW maintained at 1 or 0.4, respectively, by the robot during the seed filling stage). To study the impact of stress on TKW, the model below was used to identify associated genetic markers:

$${TKW}_{i}=\gamma_{i}+\alpha_{i}SFTSW+\beta SFTSW$$

where *TKW_i_* is the TKW for the *i*th genotype, SFTSW is an indicator of the total water deficit during the trial (integration of 1 - FTSW), γ*_i_* is the genotypic effect associated with the *i*th genotype plus the residual error, α*_i_* is the coefficient of the stress response associated with the *i*th genotype, and the β coefficient corresponds to the average response of TKW to SFTSW. We performed association test on two traits, later referred as the interaction effect (α*_i_*) and the total genetic effect (γ*_i_* + α*_i_*).

Association tests were performed using a set of 2,240 independent markers corresponding to the individual genetic bins discriminated in the 84 RILs used in this study. These markers come from SNPs detected by genomic re-sequencing and implemented on an Affymetrix AXIOM genotyping array. All individuals were previously genotyped using this array, which comprised 586,986 markers, and the results were presented by Badouin et al. (2017). A total of 55,951 markers were used in the present study, which were homozygous in SF193 (less than 10% heterozygosity or missing data) and heterozygous in SF326 × SF193 (marker frequency between 40 and 60%). Carthagene (Givry et al., 2004) was used to identify 2,240 merged markers assigned to 17 linkage groups, which were positioned on the sunflower genetic map (Badouin et al., 2017).

The association tests were performed using a multi-locus mixed-model (MLMM) proposed by Segura et al. (2012). This model is based on a classic genome wide association study (GWAS) model (Yu et al., 2006), in which marker selection is performed using a forward stepwise approach. At each step, the SNP with the smallest *p*-value is added to the model, and the *p*-values, as the residual variances, are re-estimated for all cofactors. The forward selection analysis stops when the proportion of variance explained by the model is close to zero. The analysis was conducted using the MLMM code written by Segura et al. (2012) and the ASReml-R package (Butler et al., 2009), with modifications provided by Bonnafous et al. (2018). The method used is available on CRAN in the *mlmm.gwas* package.

### Transcriptomics

A transcriptomic analysis was conducted on the 13HP02 trial plants to identify the candidate genes underlying the previously identified quantitative trait loci (QTLs). The analysis compared the transcriptomes of the SF193 × SF326 hybrid and the SF193 parental line grown under two conditions, irrigated or non-irrigated, as these genetic and stress conditions correspond to the situation studied in the genetic study using the 13HP05 trial. The transcriptome analysis was performed on plant pairs (stress-control), when the FTSW of the stressed individual reached 0.1.

Leaves were harvested between 11:00 and 13:00 h. The total number of leaves was estimated, and leaves at the position corresponding to two-thirds of the total leaf number from the bottom were tagged and termed the *n*th leaf. For the transcriptomic studies, the leaf at position *n* + 1 was used.

The leaf blades were detached from their petiole and immediately frozen in liquid nitrogen. The samples were ground using a ZM200 grinder (Retsch, Haan, Germany) with a 0.5-mm sieve. Total RNA was extracted using the QIAzol Lysis Reagent (Qiagen, Hilden, Germany), following the manufacturer’s instructions. The RNA was checked on an agarose gel using electrophoresis, and its quality and quantity were assessed using the Agilent RNA 6000 Nano Kit (Agilent Technologies, Santa Clara, CA, United States). Paired-end libraries were generated using the TruSeq sample preparation kit (Illumina, San Diego, CA, United States) according to manufacturer’s instructions, and were sequenced (2 × 100 bp, oriented) on an Illumina HiSeq 2000 by DNAVision (Charleroi, Belgium).

The transcriptomic study was performed using the EdgeR package version 3.16.5 (Robinson et al., 2010) on R version 3.3.3 (Another Canoe) (R Core Team, 2017). A gene was considered to be expressed if it was detected with at least two counts per million (CPM) in three libraries out of a larger experimental design including 142 samples. This larger experiment included 142 plants (two died prematurely) representing 24 genotypes, two water statuses and three replicates. The CPM values were computed with the cpm function in the edgeR package. A total of 27,279 genes were found to have a CPM of at least two in three libraries. Count normalization was performed as described in the edgeR user guide, using the TMM method (trimmed mean normalization) and the calcNormFactors function. Unless otherwise stated, the subsequent computation and analyses were performed on the filtered and normalized values. The experimental data and design are summarized in the data paper of Blanchet et al. (2018).

To identify differentially expressed genes (DEGs) between SF193 and SF193 × SF326, the generalized linear model pipeline in edgeR was used as described in the user manual. Contrasts were created using the makeContrasts function, with the genotypic interaction calculated as: “(SF193 × SF326.stress - SF193 × SF326.ctrl) - (SF193.stress - SF193.control).” The *p*-values were corrected using the false discovery rate method, with a cutoff of 0.05.

### Crop Modeling and Simulation

#### Model Parameterization and Evaluation

Crop modeling and simulations were used to predict the grain yield of genotypes phenotyped in the Heliaphen platform in non-observed field environments. SUNFLO is a process-based simulation model for sunflower that was developed to simulate grain yield and oil concentration as a function of time, environment (soil and climate), and crop management practice including cultivars (Casadebaig et al., 2011; Lecoeur et al., 2011). Predictions made using the model are restricted to obtainable yield (Van Ittersum and Rabbinge, 1997), and only the major limiting abiotic factors (temperature, light, water, and nitrogen) are included in the algorithm.

The model simulates the main soil and plant functions: root growth, soil water and nitrogen content, plant transpiration and nitrogen uptake, LE and senescence, and biomass accumulation. Globally, the SUNFLO crop model has about 50 equations and 64 parameters: 43 plant-related traits, among which eight are genotype-dependent, and 21 environment-related parameters. The equations and parameters used in the model are summarized in the supplementary information provided by Picheny et al. (2017). The source code is available on the INRA software repository.^1^ The INRA VLE-RECORD software environment (Quesnel et al., 2009; Bergez et al., 2013) was used as the simulation platform.

The model inputs are split into four categories: genotype, climate and soil, management actions, and initial conditions. In the model, a genotype is represented by a combination of eight genotype-dependent parameters whose values are assumed to be constant among environments, thus mimicking genetic information (Boote et al., 2003; Jeuffroy et al., 2013). All but two of the parameters were directly measured in the field trials, while the responses of LE and stomatal conductance to water deficit necessitated controlled-condition experiments (methods described by Casadebaig et al., 2016a). In this study, the measurement protocol for the Heliaphen platform was the same as in the greenhouse experiments (Casadebaig et al., 2008). The aim was to evaluate whether this new phenotyping platform could affect the prediction accuracy of the simulation model.

Four climatic variables were used as daily inputs for the simulation: mean air temperature (°C at 2 m above the ground), global incident radiation (MJ.m^-2^), potential evapotranspiration (mm, estimated using the Penman–Monteith equation), and precipitation (mm). The properties of the soil were defined by its texture, depth, and mineralization, while the initial soil conditions were defined by the residual nitrogen level and initial water content. The management practices accounted for included sowing date, planting density, irrigation, and nitrogen fertilization.

The SUNFLO simulation model was evaluated using both specific research trials (40 trials, 110 plots) and agricultural extension trials that were representative of its targeted use (96 trials, 888 plots). Using these two datasets, the model simulated the significant genotype × environment interactions and ranked the performance of the genotypes (Casadebaig et al., 2011, 2016a). The prediction error for grain yield was 15.7% when estimated across all data (9–30% in individual trials). From these two evaluations, the model was considered accurate enough to discriminate between two given genotypes and be used in simulation studies.

#### Impact of Parameterization Method on Model Accuracy

The impact on model accuracy was evaluated when using the Heliaphen phenotyping platform to estimate the value of two genotype-dependent parameters; the response of LE and plant transpiration to water deficit. In the parameterization dataset, three genotypes were common between the two field experiments and six glasshouse experiments (Casadebaig et al., 2008). The test dataset was a subset of these three genotypes from a multi-environmental network (described in Section “Plant Material, Experiments, and Multi-Environment Trials”), for which grain yield was observed in 10 locations. The prediction capacity of the model was assessed using the bias, root-mean-squared errors (RMSE), and relative RMSE (rRMSE) values, which are different metrics commonly used to evaluate the predicting ability of models. The prediction capacity of the model was compared when using the test datasets for two sets of simulations: one where genotypes were parameterized using the reference method (greenhouse experiments) and one with parameters measured using the Heliaphen phenotyping platform.

#### Simulation of Genotypes in a European Trial Network

A factorial design was created using four commercial hybrids with contrasting response traits, 42 locations sampled from sunflower cropping areas in continental Europe, and 21 years of historical climate data. The genotypes were selected according to their contrasting regulation of LE and transpiration in response to water stress, based on data from trials 14HP09 and 15HP04 (Table 1). The following genotypes were selected: MAS86OL (rapid transpiration and LE responses to water stress), LG5450HO (rapid transpiration response and slow LE response to water stress), MAS89M (slow transpiration response and rapid LE response to stress), and SY EXPLORER (slow transpiration and LE responses to water stress).

Soil property data were obtained from a local soil analysis in 13 of the 42 trials, and completed using the European soil database (ESDAC^2^; Panagos et al., 2012; Hiederer, 2013). The climatic dataset was obtained from the Agri4cast data portal (Gridded Agro-Meteorological Data in Europe). For each location considered in the study, meteorological data from 1996 to 2016 from the nearest grid point (25 km × 25 km) were used.

For each genotype × location × year combination (*n* = 3528), six output variables were simulated in the crop model: seed yield at harvest, oil content at harvest, and four time-series estimates of the impact of the selected abiotic stress (water stress, nitrogen deficiency, low temperature stress, or high temperature stress) on the simulated photosynthesis rate.

#### Clustering of Environmental Time Series

To group environments (location × year combinations) with similar abiotic stress patterns, each simulated time series was summarized by integrating the value of the considered abiotic stress variable over the crop cycle, thus defining a vector of four stress indicators. The water stress indicator was described by the integration of the fraction of transpirable soil water over time (SFTSW). The nitrogen deficiency indicator was defined by the integration of the nitrogen nutrition index (SNNI; Lemaire and Meynard, 1997). The low and high temperature stress indicators (SLT and SHT) were defined by the integration of the photosynthesis response curve to temperature over time. The HCPC function of factomineR (Husson et al., 2010) was then used to make a hierarchical classification of environments based on the simulated stress indicators (3528 environments × 4 indicators).

## Results

### Phenotyping for Plant Breeding

#### Drought Stress Management Using Heliaphen

**FIGURE 3:**
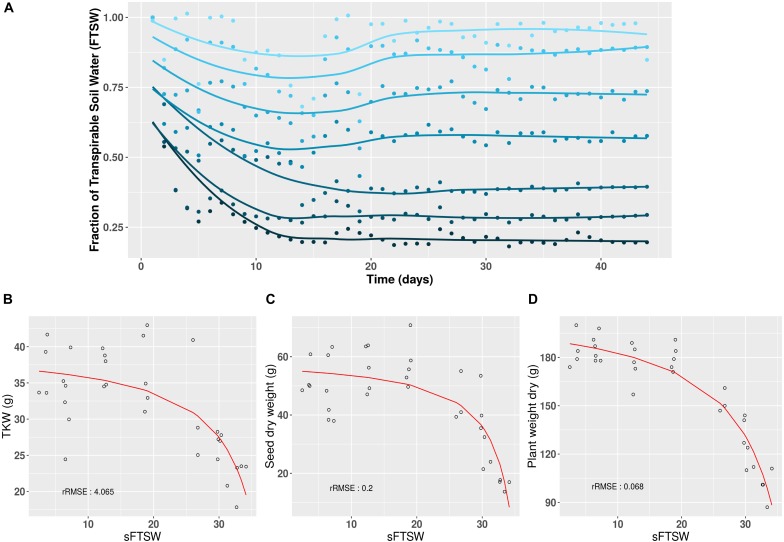
Long-term daily monitoring of water status and its impact on yield and biomass traits using the Heliaphen platform. **(A)** After anthesis (day 0), sunflower plants (NK KONDI genotype) were exposed to different water deficit levels, expressed as different fractions of transpirable soil water (FTSW; dark to light blue corresponding respectively to FTSW values from 0.2 to 1). Points correspond to the average FTSW values for six plants per stress treatment, and lines correspond to the fit of the points for each stress, calculated using the LOESS regression method. **(B)** Thousand-kernel weight (TKW) in response to changes in SFTSW (integration of 1 – FTSW) for each plant. **(C)** Seed weight (per plant) in response to changes in SFTSW. **(D)** Biomass in response to changes in SFTSW. rRMSE, relative root mean square error of the polynomial ratio model.

The aim of creating the Heliaphen platform was to precisely control the level and duration of water deficit experienced by plants at different stages of the crop cycle. Using the 16HP07 trial investigating the response of plants to water stress as an example, the Heliaphen platform was successfully used to apply different stress levels to sunflower plants of the commercial genotype NK KONDI over 40 days, from anthesis to harvest (Figure 3A). The impacts of the integrated drought stress indicator (SFTSW) on the TKW value, seed weight, and plant biomass are illustrated in Figures 3B–D, respectively. When modeling the phenotype response to water deficit using the model $Y=\frac{\left(a+bSFTSW\right)}{\left(1+cSFTSW\right)}$, the rRMSE values were 4.06, 0.20, and 0.07%, respectively.

#### Comparison Between Phenotypes Observed Using the Heliaphen Platform and in the Field

**FIGURE 4:**
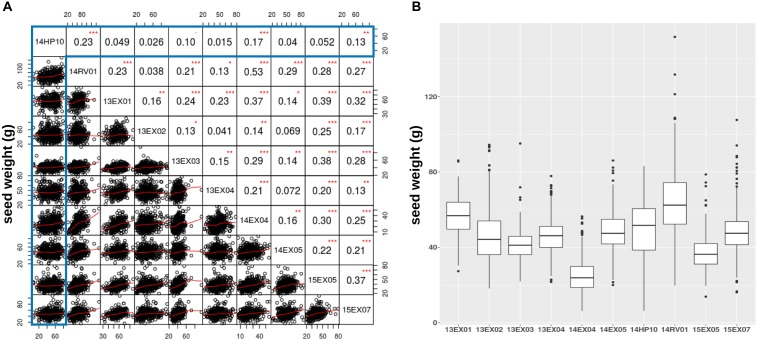
Comparison of seed weights per plant measured on the Heliaphen phenotyping platform and in field conditions. **(A)** Correlation matrix between the seed dry weights of the SUNRISE hybrid panel (*n* = 426) grown on the platform (14HP10, highlighted in the gray box) and the nine field trials (14RV01, 13EX01–04, 14EX04–05, 15EX05, and 15EX07). The correlation coefficients are indicated in the upper right section of the panel, with significant differences indicated by asterisks (^∗^*p* < 0.05; ^∗∗^*p* < 0.01; ^∗∗∗^*p* < 0.001). **(B)** Boxplot of the seed dry weights observed for the SUNRISE panel.

We also evaluated how traits measured on the Heliaphen platform correlated to those observed in field conditions. For this purpose, we compared the weight of seeds produced by each plant grown in the trials conducted on the Heliaphen platform (14HP10 trial) and in the field (14RV01 trial). The plants used in both trials shared the same genetic material (SUNRISE hybrid genotype panel), growing period, and environmental conditions. The seed weight measurements between the two tests were significantly correlated (*R* = 0.23, *p*-value < 0.001; Figure 4A). This population was also evaluated in eight other trials in a European network over 3 years (trials 13EX01–04, 14EX04–05, 15EX05, and 15EX07). The average correlation between the field trials was 0.22 (Figure 4B), which was similar to the correlation level found between platform and field trials grown concurrently (14HP10 and 14RV01). The Heliaphen trial was also significantly correlated to two other field trials [14EX04 (*R* = 0.17) and 15EX07 (*R* = 0.13)].

Figure 4B shows that, although the pedoclimate has a strong impact on the seed production of the sunflower panel, resulting in significant differences between the variances and means of the different trials, the seed weight distribution measured on the platform is within the same range as the field trials.

#### Association Genetic Analysis of the Phenotypes Measured on the Heliaphen Platform

**FIGURE 5:**
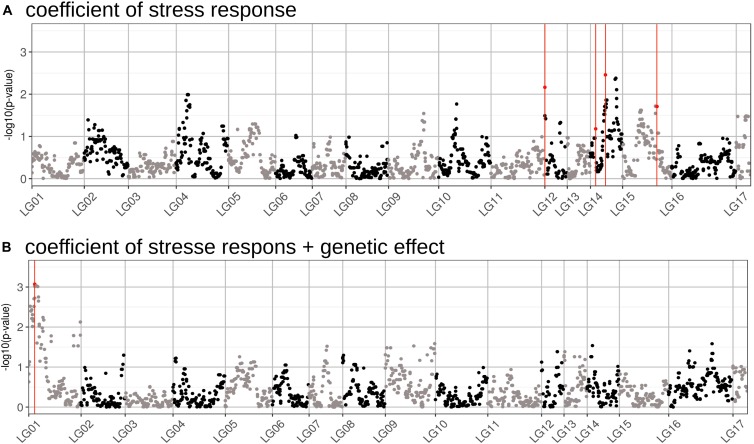
Manhattan plots of the GWAS performed for two traits related to drought stress tolerance. **(A)** Results of the GWAS performed on the genetic coefficient of stress response. **(B)** Results of the GWAS performed on the sum of the coefficients for the stress response and the genotypic effect (total genetic effect). The markers found to be associated with each effect using the MLMM method are highlighted in red. The *p*-values on the graph correspond to those obtained using a classical GWAS model, performed during the first step of the MLMM method.

We performed a GWAS on the response of seed weight to a constant water deficit during the reproductive phase (FTSW = 0.4) in a population of RILs (13HP05 trial). The genetic markers associated with the TKW response to drought stress were modeled using the linear model described in Section “Genetic Association Study” of Section “Materials and Methods.” In order to identify markers associated with this response, an association test (MLMM) was performed on the genetic coefficient of the stress response (*α_i_*, interaction effect) and on the sum between the stress response coefficient and the genetic effect (*γ_i_* + *α_i_*, total genetic effect). Using this approach, we selected a set of markers that together explained the genetic variance of the studied characters. Four markers were found to be associated with the coefficient of the response to drought stress and one to the sum of the stress response coefficient and the genetic effect (Table 2). All markers were positioned on the sunflower genetic map; the Manhattan plots in Figure 5 enable the visualization of the *p*-values calculated for the first MLMM step corresponding to the classical model of GWAS (Yu et al., 2006).

**Table 2:** Markers associated with the thousand-kernel weight response to drought stress, and the nearby candidate genes with differential expression in droughted and irrigated plants of the RIL population parents.

Trait	Marker	LG	Position (bp)	Effect	Gene	Distance (bp)
α*_i_*	AX-105774006	12	164538019	0.08	HanXRQChr12g0385481	1258962
α*_i_*	AX-105362253	14	123649962	0.12	HanXRQChr14g0445121	1443617
α*_i_*	AX-105376351	14	167871915	-0.11	HanXRQChr14g0459681	20774
α*_i_*	AX-105568999	15	92795704	0.08	HanXRQChr15g0486311	2980461
α*_i_* + γ*_i_*	AX-105798220	1	61598886	2.74	HanXRQChr01g0009921	4628392


*Trait column is based on the modeled response of thousand-kernel weight to water stress with the traits used in association defined as the interaction effect (α*_i_*) and the total genetic effect (γ*_i_* + α*_i_*). Positions, names, and distances are based on the HanXRQv1 genome.*

#### Transcriptomic Analysis

**FIGURE 6:**
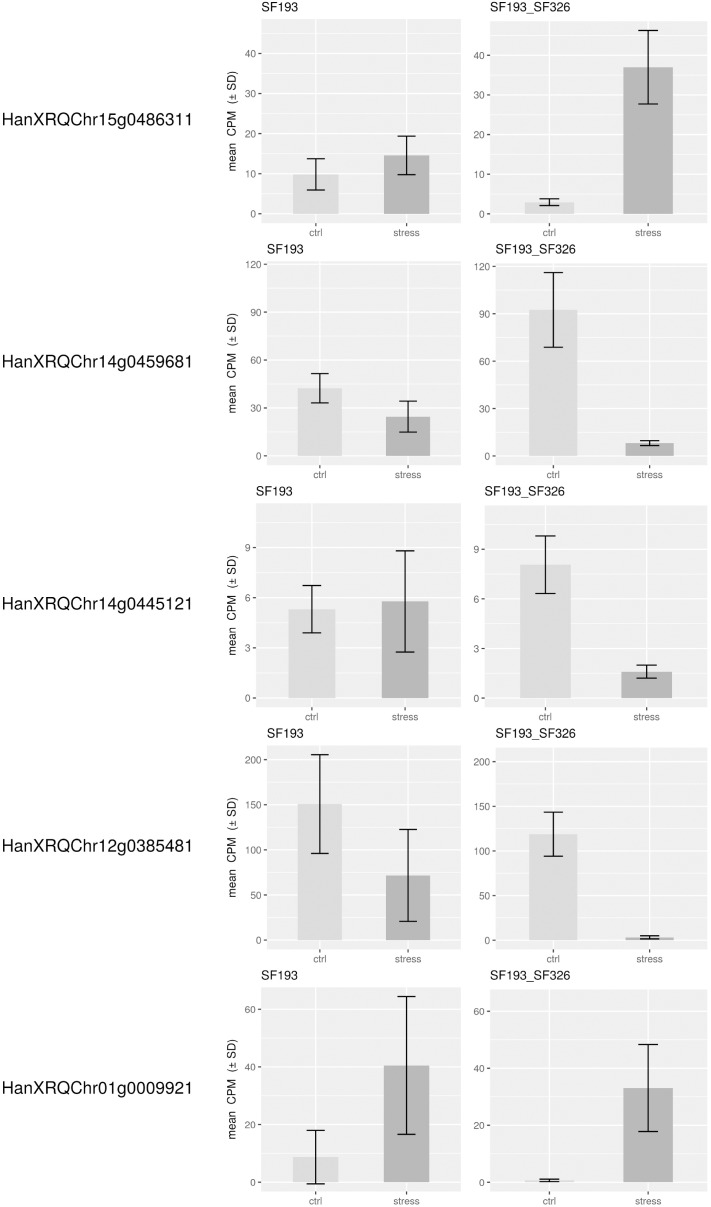
Expression levels of genes in close proximity to SNPs associated with the stress response. Expression levels of differentially expressed genes within 5 Mb of the SNPs associated with the drought stress response in the backcrossed RIL population [SF193 × (SF193 × SF326)]. Expression levels (count per million, CPM) were evaluated in SF193 and the SF193 × SF326 plants grown under water stress (stress, dark gray) or normal irrigation (control, light gray), with three replicates.

In order to identify the best candidate genes carrying the polymorphisms responsible for the differing responses to the water deficit, we studied gene expression in both the homozygote SF193 and the heterozygous SF193/SF326 backgrounds, the same genotypes used for the QTL detection. The transcriptomic analysis revealed that on the 27,278 expressed genes, 1284 are differentially expressed in interaction (Genotype × Treatment) and 555 and 1659 are, respectively, differentially expressed in SF193 and SF193 × SF326 as a function of the treatment conditions (Supplementary Data Sheet S1 in Supplementary Material). An analysis of enrichment in gene ontology terms (GO) was performed using the approach proposed by AgriGO (Du et al., 2010) on the sets of DEGs. Results are available in Supplementary Material (Supplementary Data Sheet S2–S4 and Supplementary Figures S1–S8 in Supplementary Material). The pathways identified in response to drought in our study correspond to those previously described by Rengel et al. (2012) such as auxin signaling, response to abiotic stimulus, cell wall organization, and biogenesis as well as water transport processes (Kiani et al., 2007). Based on the hypothesis that the causal polymorphism would influence the expression of the candidate gene, we searched for genes around the associated markers (±5 Mb) whose expression levels showed significant interaction (FDR < 0.05) between drought stress and genotype in the transcriptomic experiment (13HP02 trial). The expressions of the most significant genes in those regions are shown in Figure 6.

On chromosome 12, we identified a marker significantly associated with the drought stress coefficient. The effect of the alternative allele in SF326 on the coefficient of the stress response was 0.084, providing this genotype with drought tolerance. We were able to identify the gene HanXRQChr12g0385481, situated about 1.2 Mb upstream of the marker. The protein encoded by this gene shares sequence similarity (54% of protein identity) with the *Arabidopsis thaliana* protein UDP-GLYCOSYLTRANSFERASE 90A1 (UGT90A1). The UDP glycosyltransferases are a large protein family, some of which are involved in abiotic stress (including drought) responses in *Arabidopsis* (Li et al., 2016a,b, 2017).

We identified two markers associated with the drought stress coefficient on chromosome 14. The effects of the alternative alleles in SF326 on the coefficient of the stress response were 0.118 and -0.106, providing tolerance and sensitivity, respectively. We identified the gene HanXRQChr14g0445121 located 1.4 Mb upstream of one of the markers. This product of this gene shared 47.71% of the sequence of the *Arabidopsis* protein RING-H2 FINGER PROTEIN ATL54, which is involved in the formation of the secondary cell wall (Noda et al., 2013). The second gene identified on chromosome 14 was HanXRQChr14g0459681, situated 20 kb upstream of the other marker. This gene encodes a protein that shares 38.45% of its identity with the *Arabidopsis* protein SMAX1-LIKE 7 (SMXL7), which is involved in the regulation of shoot development (Liang et al., 2016).

On chromosome 15, one marker was found to be associated with the drought stress coefficient. The effect of the alternative allele in SF326 on the coefficient of the stress response was 0.085, providing tolerance. The gene HanXRQChr15g0486311 was found to be 2.9 Mb upstream of this marker. Its product shares 52.81 and 46.80% of its sequence with the *Arabidopsis* transcription factors TCP19 and TCP9, respectively. These transcription factors are involved in the control of leaf senescence and the regulation of jasmonic acid (Danisman et al., 2012, 2013), processes which have been widely reported to be linked to the drought stress response in many different species, including sunflower (Marchand, 2014; Andrade et al., 2017).

One marker on chromosome 1 was associated with the total genetic effect (sum of the drought stress genetic coefficient and the genetic effect). The effect of the alternative SF326 allele on the total genetic effect was 2.737, providing drought tolerance to this genotype. We identified the gene HanXRQChr01g0009921, situated 4.6 Mb upstream of this marker, which encodes a protein sharing 66.47% of its sequence with the monofunctional riboflavin biosynthesis protein RIBA3 in *Arabidopsis*. This protein is involved in the biosynthesis of riboflavin (Hiltunen et al., 2012), which is believed to alleviate ROS production during osmotic stress (Deng et al., 2013).

The Heliaphen platform allowed us to successfully identify genomic regions associated with the water deficit stress response affecting yield traits. We could compare these data with other studies to identify candidate genes putatively involved in the response to drought stress. Further studies are required to elucidate the involvement of these candidate genes in the changes in yield-related traits caused by water stress, but the platform was instrumental in generating these promising results.

### Phenotyping for Crop Modeling

#### Impact of Parameterization Method on Crop Model Accuracy

The SUNFLO crop model (Casadebaig et al., 2011) uses phenotypic traits as genotype-dependent input parameters. Two of these describe the response of LE and transpiration to water stress, requiring dynamic leaf area measurements and the assessment of water deficit at the plant level, respectively. Here, we compare how the model accuracy is affected by the measurement of these parameters using the Heliaphen platform or the reference method based on greenhouse experiments (Casadebaig et al., 2008).

**FIGURE 7:**
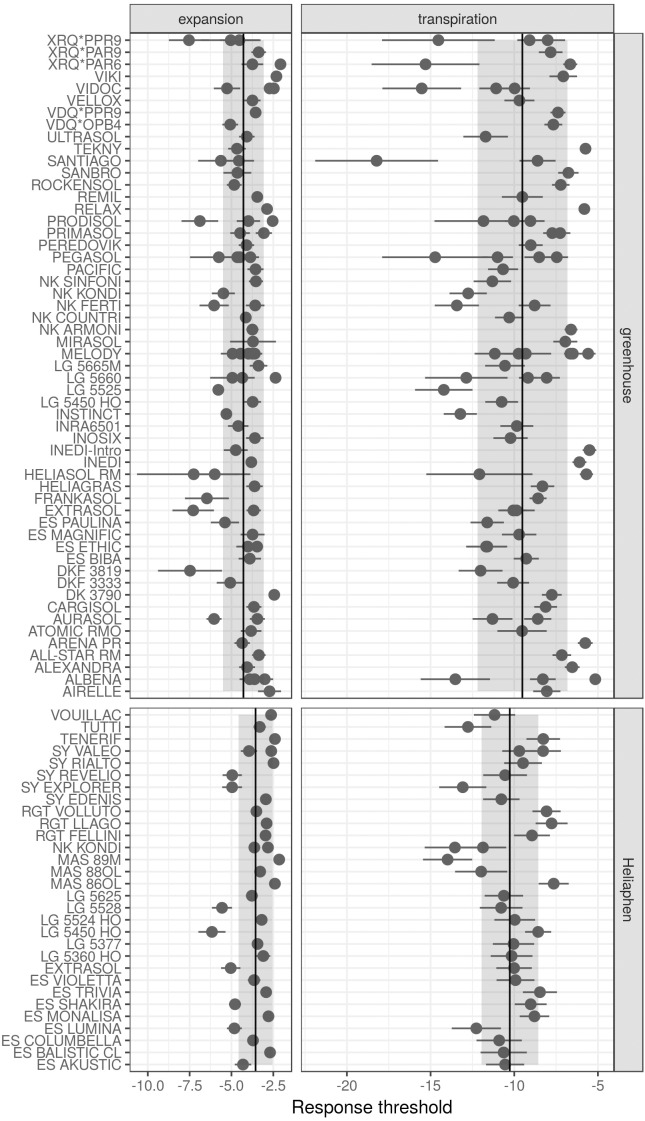
Drought response parameters of the SUNFLO crop model measured for different commercial hybrids in a greenhouse environment and using the Heliaphen platform. Points correspond to the value of the parameter for each genotype. Ranges indicated with dark lines correspond to the standard error of the estimated parameter. Ranges indicated with shading correspond to the standard deviation for the population phenotyped in the greenhouse (*n* = 32) or Heliaphen platform (*n* = 82).

We first compared the standard error of the parameter estimations performed using measurements of commercial hybrids taken in either the Heliaphen or greenhouse conditions (Figure 7). Lower parameter values indicate that the genotypes started to regulate either LE or transpiration at lower FTSW values, indicating that the genotype maintains its carbon assimilation at greater water deficits. In comparison with measurements performed on greenhouse experiments, those on the Heliaphen platform resulted in similar errors in parameter estimation for the transpiration response (∼1.1 SD for both conditions), but halved the error in the estimated LE response (0.3 SD in the Heliaphen measurements vs. 0.7 SD in the greenhouse ones). Additionally, the estimation errors in the Heliaphen measurements were not dependent on the parameter value, as was the case for the greenhouse measurements. Because these traits are used as input values in the simulation model and have an impact on yield prediction, we also evaluated how the model prediction accuracy was impacted when using different parameterization conditions. The rRMSE values for the yield prediction were equivalent (13%) between the simulations made from the data obtained from the Heliaphen platform trials and from the greenhouse trials. We can therefore conclude that the accuracy of predictions made using measurements taken using the Heliaphen platform is conserved, and that this platform facilitates greater precision in the measurement of two input parameters, LE and transpiration.

#### Integrating Traits Measured Using the Heliaphen Platform to Simulate Genotype–Environment Interactions

The Heliaphen platform enables the automated measurement of the genotype-dependent traits of plant varieties. This information can be fed into a simulation model to estimate their productivity in different types of environments. To illustrate this approach, we selected four genotypes with contrasting responses to water deficit stress (Table 3), and used a computational experiment to compare them in a trial network representative of sunflower-growing regions in Europe. The selected genotypes were simulated using the SUNFLO crop model in 42 locations over 21 climatic years, from 1996 to 2016. We defined an environment as the combination of a location and a year (42 locations × 21 years gives a total of 882 environments). Simulations were performed for each genotype in each of the environments, and the average abiotic stress indicators of the four genotypes in each environment were calculated.

**Table 3:** Response parameters of genotypes used for the simulation.

Genotype	Transpiration rate	Leaf expansion rate
LG 5450H0	-9.66 (+)	-4.94 (-)
MAS 86OL	-7.64 (+)	-2.40 (+)
MAS89M	-13.98 (-)	-2.15 (+)
SY Explorer	-13.08 (-)	-4.96 (-)


*(+) indicates and early response of the process to water stress (occurring at low water deficit level), and (-) indicates a late response (occurring at high water deficit).*

**FIGURE 8:**
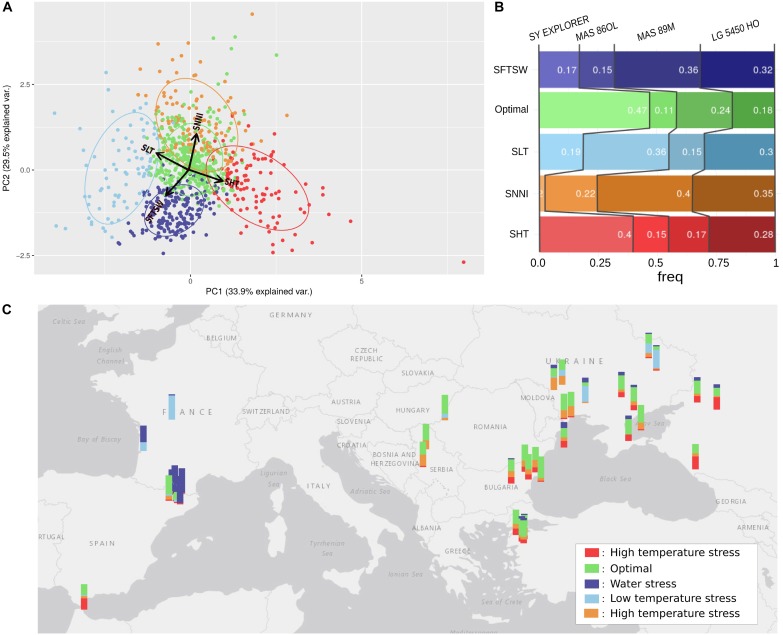
Use of simulation modeling to characterize the stresses in 42 European sunflower cropping environments. **(A)** Clustering of environments (year-location) by the principal components of average simulated stresses for four sunflower genotypes. The PCA was performed using parameters for high temperature stress (SHT), low temperature stress (SLT), water stress (SFTSW), and nitrogen deficit (SNNI). Variables are represented with red arrows. The environments are clustered into five groups; four are associated with a specific stress and named accordingly, and the one without any obvious stress influence was named “Optimal.” **(B)** Standardized yields of each genotype ranked for each simulated environment group, with the frequency of the highest ranked genotype for each environment cluster reported inside the bars. **(C)** Map of Europe with a barplot at each location. The colors of the barplots indicate the occurrence of the location in the different environment clusters.

Figure 8A depicts a principal component analysis (PCA) in which each point depicts the average stress indicators in one environment. Five environment clusters were defined, and each group (or environment type; Chenu et al., 2013) was labeled following the projection of stress indicators in the PCA plane. For each environment types, the frequency for which each genotype ranked the most highly is represented in Figure 8B. Finally, the relative frequencies of each environment type could then be mapped to the corresponding geographical locations (Figure 8C) to illustrate the most frequent abiotic stress factors in the different sunflower-growing regions across Europe; for example, the high frequency of drought stress in France could be explained by the impact of the shallower soil depths in these locations.

We also aimed to identify environment types where specific genotypes would perform better than others, and therefore adjusted the simulated yields by the average yield of each genotype and ranked the genotypes on this index for each environment (Figure 8B). Thus, we could characterize the abiotic stress tolerance profiles of each genotype, revealing the types of environment that maximize the yield of each genotype in comparison with the others. Based on this method, we revealed that LG5450HO displayed a balanced tolerance profile (relatively good yields in each environment type), SY EXPLORER was particularly adapted to heat-stressed environments while MAS86OL was more adapted to cold-stressed environments, and MAS89M was adapted to nitrogen stress and water-stressed environments. The Heliaphen platform could therefore provide key phenotyping information for these sunflower genotypes, which would enable us to better select cultivars suited to a particular environment and identify the areas where they could potentially perform best.

## Discussion

In order to characterize sunflower genotypes for their response to drought stress, we developed the Heliaphen high-throughput phenotyping platform. This facility allows different water stress scenarios to be applied to plants, and the subsequent evaluation of the effects of these stresses on the overall sunflower yield and its component traits. Results obtained on the platform confirmed its reliability in implementing water stress on large panels of plants, and its automatic measurement of specific plant traits for use as input parameters in crop simulation models.

The Heliaphen platform is a unique automated phenotyping facility. Most phenotyping platforms rely on strongly controlled environmental conditions and are operated inside greenhouse or growth chambers, and mainly target the vegetative part of the plant cycle because of the space required by mature crop plants (Granier et al., 2005; Pereyra-Irujo et al., 2012; Honsdorf et al., 2014; Cabrera-Bosquet et al., 2016). These facilities make it possible to control the biotic and abiotic environment more finely but consequently move away from the more variable light spectra and climatic conditions in the field. By contrast, phenotyping tools such as unmanned aerial vehicles (Sankaran et al., 2015; Hu et al., 2018) or robots (Deswarte et al., 2015; Underwood et al., 2017) can measure plants in actual agricultural conditions, although with little control over climate scenarios. The Heliaphen platform provides a more realistic environment than other automated phenotyping platforms within glasshouses, while still enabling climate factors such as water and nutrient availability to be tightly controlled.

Automated phenotyping tools for the Heliaphen platform are continuously under development. Different sensor types are currently being developed to monitor new sunflower traits; for example, ultrasonic sensors to monitor plant height, laser scanners to measure the stem diameter, and a light curtain sensor (Rapidoscan RS-C-025-768-ECT) to phenotype the leaf area profile. The analysis of the growth of organs (stem, petiole, and leaf blade) over time has been greatly improved by the development of an image analysis algorithm, in which a 3D point cloud is computed using structure from motion techniques based on RGB images of the plant. The 3D cloud is then segmented into organs whose area, length, and volume can be estimated (Gélard et al., 2017). In addition to sunflower, we have successfully used the platform to monitor the growth and development of other species such as soybean, maize, and tomato. It would also be possible to conduct trials combining several stresses, for example, to study how plant responses to nutrient stress interact with their response to a water deficit.

We first aimed to validate the possibility of employing specific drought scenarios and comparing the responses of yield-related traits assessed on the platform to field conditions. We successfully applied different stress intensities over various time scales, including the entire grain-filling period (about 40 days). In addition, the comparison of seed production between field trials and a Heliaphen platform trial showed a relatively poor but significant (because of the power of the statistical design) correlation between the observed seed weights. This highlights the importance of genotype–environment interactions and the difficulty of predicting them and therefore to identify stable QTLs across environment scenarios. Meanwhile, we can also consider that this correlation indicates the potential of the Heliaphen facility to produce phenotypes relevant to agronomic conditions in the field. It is therefore possible to study and identify genes putatively controlling drought tolerance in yield traits using the Heliaphen platform.

### Identifying Genes Controlling Drought Tolerance in Sunflower Yields

We subjected a RIL population to different drought stress scenarios and conducted a genetic study, identifying a total of five genomic regions associated with the response of seed weight to water stress. The transcriptomes of both the homozygous line SF193 and the heterozygous SF193/SF326 hybrid were assessed in both stressed and unstressed conditions. Following the hypothesis that the causal polymorphisms would influence gene expression, we looked for the most significant DEGs in the region surrounding the identified markers.

Among the identified genes, several stood out as potential drought-response candidate genes. One encodes a protein belonging to the large UDP glycosyltransferase family, some of which are involved in the responses to abiotic stress (including drought) in *Arabidopsis* (Li et al., 2016a,b, 2017). We also detected a putative TCP transcription factor homologous to AtTCP19, which is reported to be involved in the control of leaf senescence and the regulation of jasmonic acid metabolism in *Arabidopsis* (Danisman et al., 2012, 2013). The TCP transcription factors have been linked to the drought responses of many plant species, including sunflower (Marchand, 2014; Andrade et al., 2017). Another candidate gene encodes a protein homologous to riboflavin RIBA3. In *Arabidopsis*, this protein is involved in the biosynthesis of riboflavin (Hiltunen et al., 2012), which is suggested to relieve the production of ROS during osmotic stress (Deng et al., 2013).

In our study, the distances between the identified genes and the positions of the markers were sometimes very large; for example, the marker detected on chromosome 1 is more than 4 Mb away from the putative candidate gene identified. The association markers represent genomic regions between two recombination points, so it is not surprising to have a large distance between the markers and the DEGs. Extra care must be taken when selecting candidate genes at a relatively large distance from the genetic marker of a trait; however, selecting a gene whose expression regulation by drought stress is different in the two parental backgrounds and is positioned near the polymorphic marker associated with the drought response does constitute an efficient method by which to identify a small number of relevant genes of interest.

The Heliaphen platform allowed us to identify genomic regions associated with the water deficit stress response affecting yield traits, that we must consider with great care given the small size of the population and the reduced statistical power of the association analysis. Future studies should aim to identify the genomic regions associated with the physiological or developmental responses to drought stress in other organs or over alternative growth periods, such as the vegetative stage or in the developing seeds, and combine this with a transcriptomic analysis to identify the responsible genes. The Heliaphen platform can also be used for functional studies, such as broad transcriptomic analyses (Badouin et al., 2017) to identify DEGs under water stress conditions and other omics techniques combined with manual descriptions of classical physiological traits, such as photosynthesis, transpiration, and osmotic adjustment.

### Using Simulation to Predict Genotype-Environment Interactions

The other major goal of developing the platform was to integrate ecophysiology and crop modeling. Physiological traits such as water deficit levels, leaf area, and water loss dynamics were more precisely measured in the Heliaphen platform than in the greenhouse and could be successfully used as parameters in a crop simulation model. The accuracy of the SUNFLO model was similar using the reference measurements taken in the greenhouse or those obtained using the platform. The platform setup is easier and more efficient than making manual greenhouse measurements, and therefore facilitates the evaluation of stress response traits in a large number of genotypes each year, keeping pace with the development of new varieties.

For the sunflower crop, while low-throughput phenotyping enabled the link between crop physiology and modeling, we showed in this study that the automation allowed us to use functional genomics tool to better study the genetic basis of complex traits (Yin et al., 2018). While other phenotyping platforms are focused on this goal, such as the PHENOARCH platform (Cabrera-Bosquet et al., 2016) recently used by Chen et al. (2018) to estimate genotype-specific radiation use efficiency in complex canopies through reverse modeling; platforms operating in outdoor conditions are not frequent (Araus et al., 2018). The LeasyScan platform (Vadez et al., 2015), which combine 3D imaging and lysimetry, rely on a similar strategy to assess canopy traits affecting water use. Overall, whether in indoor or outdoor conditions, platforms bridging phenotyping and modeling are very recent tools and it still speculative to consider that semi controlled (as the Heliaphen platform) or controlled greenhouse environment limits the transferability of results to agricultural field conditions.

We also expanded the scope of our study by designing a simulation experiment where the phenotyped genotypes could be used to reveal abiotic stresses occurring at the plant level in a large range of environments. Similar cropping conditions could then be grouped together to identify broad environment types with more predictable stress dynamics (also known as envirotyping; Xu, 2016). Using this method, we identified the major stresses occurring in the various sampled locations. These type of environments matched the cropping conditions targeted when designing the experimental network: more optimal cropping conditions in Eastern Europe, cold-stress in continental and northern locations, and drought stress caused by shallow soils in South-West France.

The accuracy of this method depends on the quality of the data used to describe the locations, including the climate, soil properties, and crop management systems. We found that the method was particularly sensitive to the estimation of the water capacity of the soil. As finer environmental descriptions become available in the future, we hope to increase the resolution and span of the simulations, which could be used to extend the classical evaluations of new varieties and enable a better recommendation of their growing range based on the environment types identified in the simulation studies. Although the variety recommendation method performed in this study only illustrated abiotic stress tolerances and does not constitute an operational selection method for use by growers (as genotypes can greatly differ from their potential yields), this is an important step to link plant phenotyping platforms and previous model-based variety recommendations (Casadebaig et al., 2016a). In addition, model-based environmental characterization was previously shown to be a powerful technique for the genetic study of yield responses to combined abiotic stresses (Mangin et al., 2017) because simulated abiotic stresses variables were closer (more related to yield) to the plant stress, and therefore gene action, than variables derived from pedoclimatic data (Lasky et al., 2015; Rasheed et al., 2017).

A challenge for plant breeders is to predict the performance of novel genetic materials in different pedoclimatic environments and under different crop management strategies. To achieve this, predictive approaches bridging quantitative genetics and crop modeling are currently being developed to scale traits from the molecular level to the crop level (Bustos-Korts et al., 2016). One option is to process the data in two steps, first using genomic predictions to estimate the genotype-dependent parameters (traits assumed to be more heritable) of a crop simulation model and then using the model to predict performance-related traits (assumed to handle genotype-environment interactions) as a function of the environmental data [e.g., Chenu et al., 2009 for maize; Quilot-Turion et al., 2016 for peach (*Prunus persica*)]. The first step, involving the genetic analysis of the input parameters of the model either to estimate the allelic effects of a QTL or to train a genomic selection population, largely relies on phenotyping capacity (Cooper et al., 2014). Such analyses can be performed using the Heliaphen platform, which illustrates its potential for accelerating the enhancement of our understanding of the genetic control of drought stress plasticity, as well as characterizing complex response traits in a variety of genotypes. It also enables crop model-based approaches to characterizing and clustering cropping environments, leading to a better match between genotypes and their optimal growing conditions.

## Author Contributions

NB, DV, PB, DC, CC, J-FL, GT, PV, PC, and NL designed the presented idea. FG, LG, BM, PC, and NL developed the theory and performed the computations. FG, PC, and NL wrote the manuscript with input from all authors. PV, PC, and NL conceived the study and were in charge of overall direction and planning.

## Conflict of Interest Statement

The authors declare that the research was conducted in the absence of any commercial or financial relationships that could be construed as a potential conflict of interest.

## Footnotes

1 https://forgemia.inra.fr/record/sunflo.git

2 esdac.jrc.ec.europa.eu
