# Supplementary material for: Heliaphen, an Outdoor High-Throughput Phenotyping Platform for Genetic Studies and Crop Modeling
Source: Front Plant Sci. 2019 Jan 16;9:1908. doi: 10.3389/fpls.2018.01908 (PMC6343525; doi:10.3389/fpls.2018.01908)
Supplement: Supplementary file 1 [file Table_1.DOCX]

Supplementary Material

Heliaphen, an Outdoor High-Throughput Phenotyping Platform for Genetic Studies and Crop Modeling

**Florie Gosseau^1^, Nicolas Blanchet^1^, Didier Varès^1^, Philippe Burger^2^, Didier Campergue^3^, Céline Colombet^3^, Louise Gody^1^, Jean-François Liévin^3^, Brigitte Mangin^1^, Gilles Tison^3^, Patrick Vincourt^1^, Pierre Casadebaig^2§^*, Nicolas Langlade**^1§^

^1^LIPM, Université de Toulouse, INRA, CNRS, Castanet-Tolosan, France

^2^AGIR, Université de Toulouse, INRA, Castanet-Tolosan, France

^3^UE Auzeville, INRA, Castanet-Tolosan, France

*** Correspondence:** [**pierre.casadebaig@inra.fr**](mailto:pierre.casadebaig@inra.fr) **;** [**florie.gosseau@inra.fr**](mailto:florie.gosseau@inra.fr)**;**

*Supplementary Material should be uploaded separately on submission. Please include any supplementary data, figures and/or tables. All supplementary files are deposited to FigShare for permanent storage and receive a DOI.*

# Supplementary Data

## Supplementary Data 1(File: InteractionGxT_lrt_13HP02.csv)

Results of transcriptomic analysis (13HP02) to identify differentially expressed genes (DEGs) on the interaction between genotype (SF193 and SF193×SF326) and treatment (irrigate and not irrigate). Default edgeR2 outputs are presented including the p-values were corrected using the false discovery rate method, FDR column, with a cutoff of 0.05.

## Supplementary Data 2(File: Enrichment_GO_Interaction.csv)

List of GO terms enriched in the set of differentially expressed gene on the interaction (genotype x treatment) obtained by the hypergeometric test with the analysis tool of AgriGO.

## Supplementary Data 3(File: Enrichment_GO_genotype_intersection.csv)

List of GO terms enriched in differentially expressed genes in function of their genotype in both control and stress conditions obtained by the hypergeometric test with the analysis tool of AgriGO.

## Supplementary Data 4(File: Enrichment_GO_treatment_intersection.csv)

List of GO terms enriched in differentially expressed genes in function of the water stress in both SF193 and SF193xSF326 genotypes obtained by the hypergeometric test with the analysis tool of AgriGO.

# Supplementary Figures

# 3.1 Supplementary Figure 1


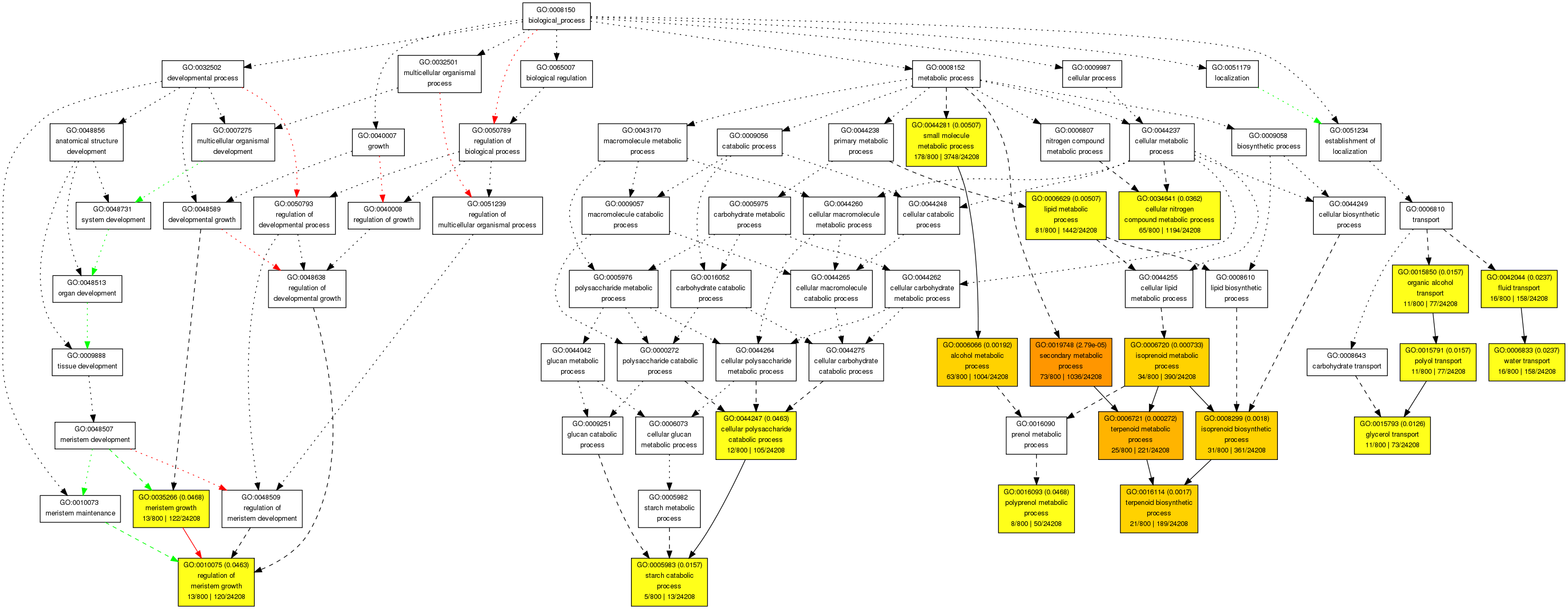
**Supplementary Figure 1.** (Graph_BP_interaction.png) Hierarchical tree graph of GO terms of biological process enriched in the set of differentially expressed gene on the interaction (genotype x treatment) obtained by the hypergeometric test with the analysis tool of AgriGO.

## 3.2 Supplementary Figure 2


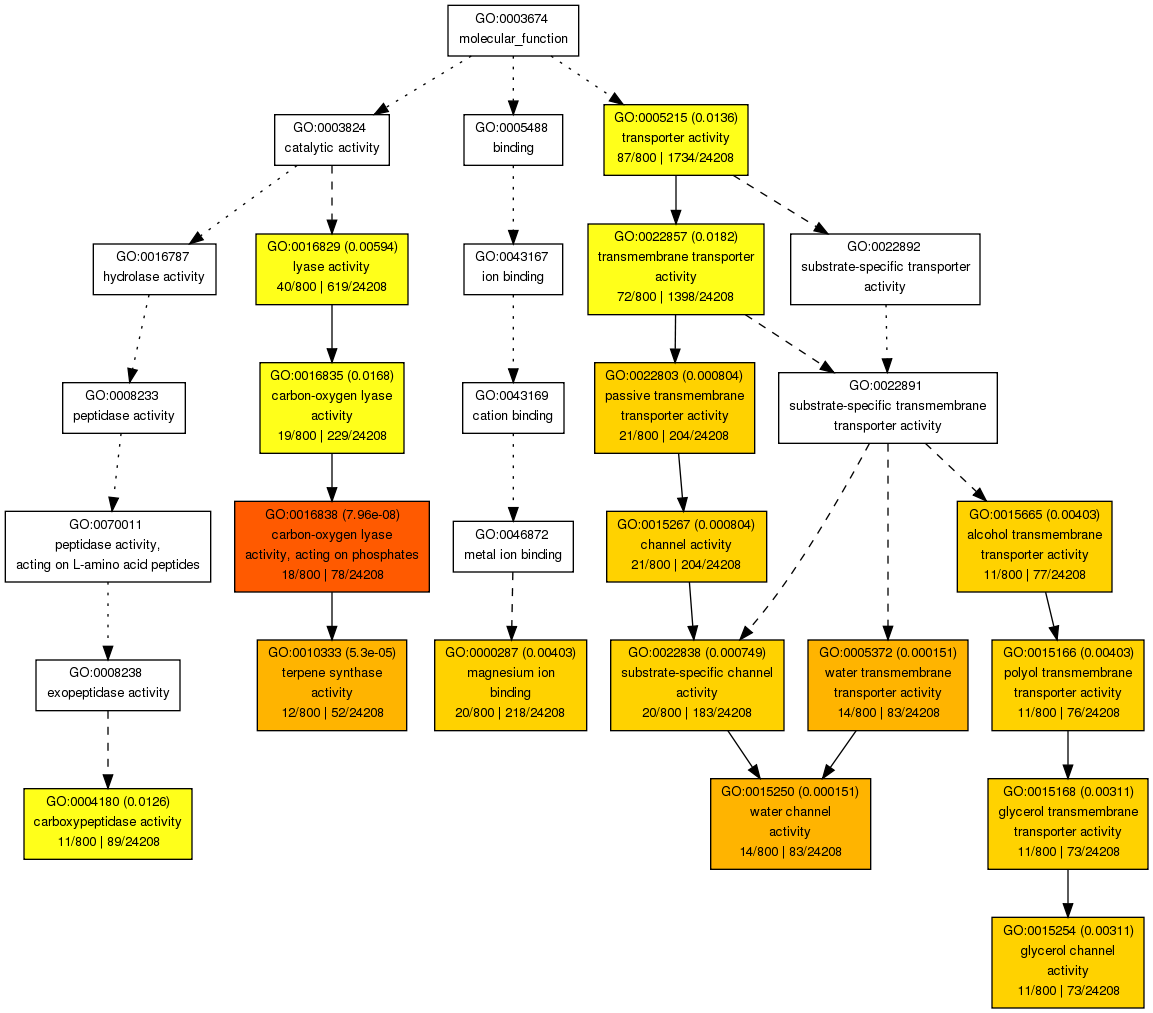
**Supplementary Figure 2.** (Graph_MF_interaction.png) Hierarchical tree graph of GO terms of molecular function enriched in the set of differentially expressed gene on the interaction (genotype x treatment) obtained by the hypergeometric test with the analysis tool of AgriGO.

## 3.3 Supplementary Figure 3


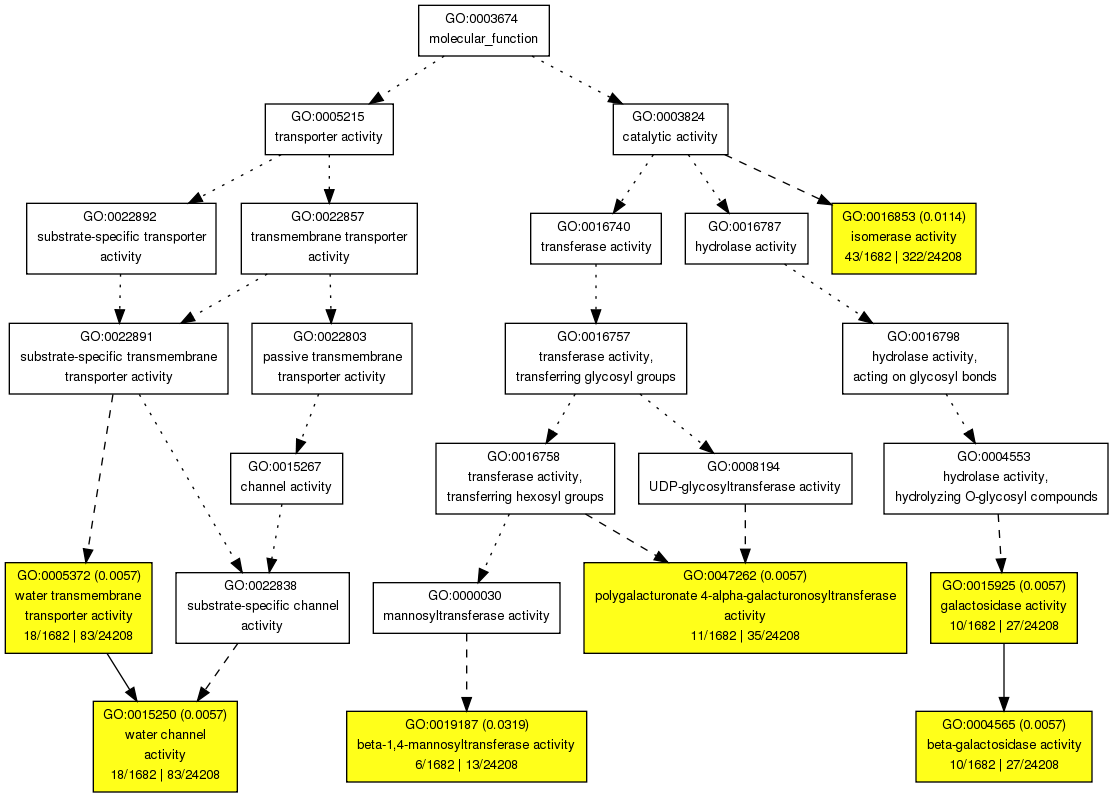
**Supplementary Figure 3.** (Graph_MF_treatement_intersection.png) Hierarchical tree graph of GO terms of molecular function enriched in differentially expressed genes in function of the water stress in both SF193 and SF193xSF326 genotypes obtained by the hypergeometric test with the analysis tool of AgriGO.

## 3.4 Supplementary Figure 4


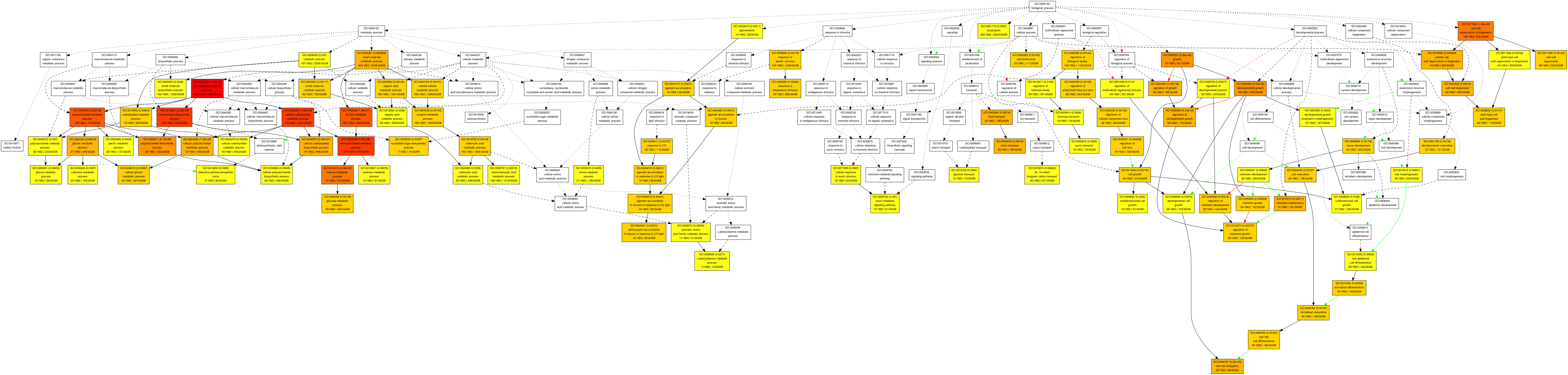
**Supplementary Figure 4.** (Graph_BP_treatement_intersection.png) Hierarchical tree graph of GO terms of biological process enriched in differentially expressed genes in function of the water stress in both SF193 and SF193xSF326 genotypes obtained by the hypergeometric test with the analysis tool of AgriGO.

## 3.5 Supplementary Figure 5


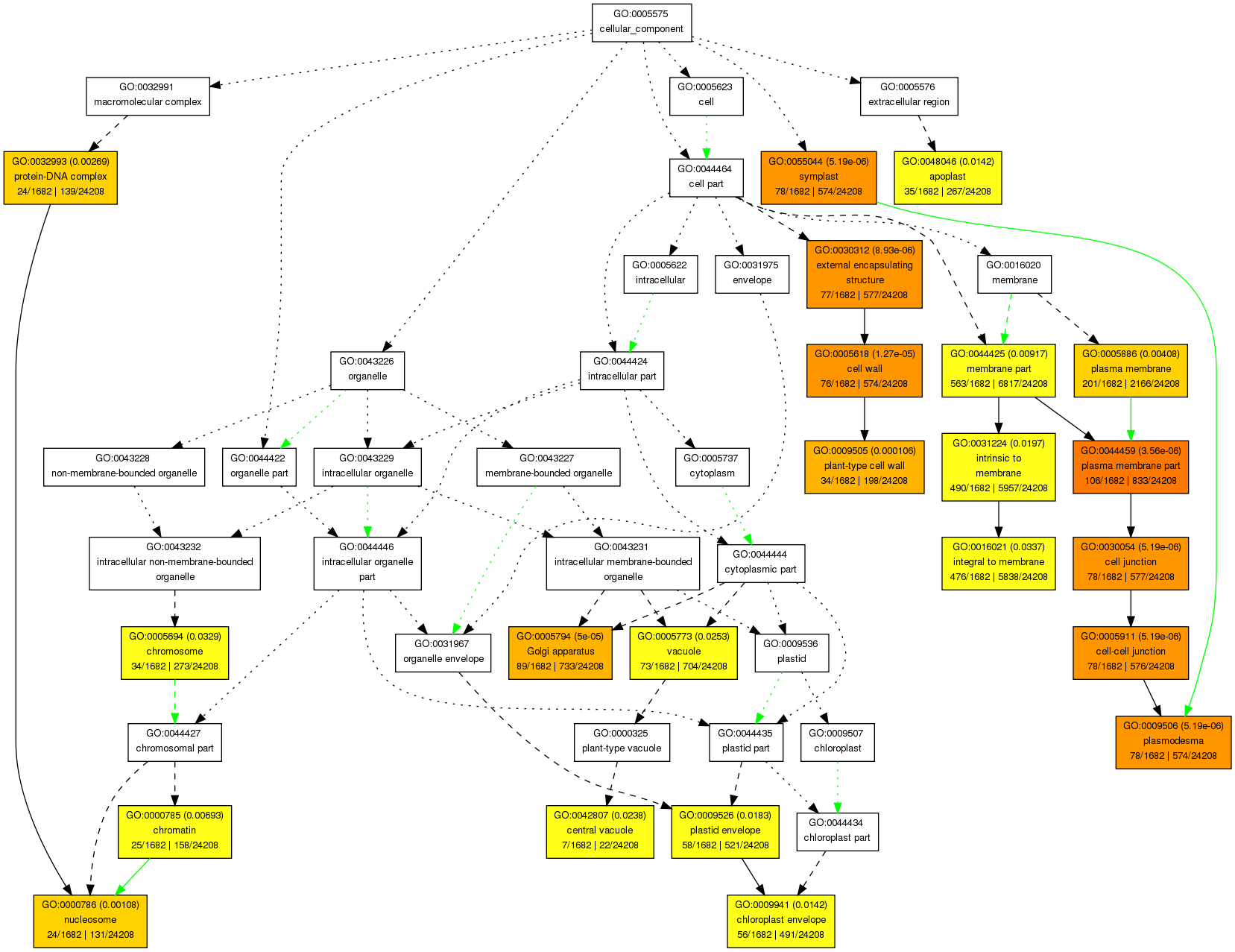
**Supplementary Figure 5.** (Graph_CC_treatement_intersection.png) Hierarchical tree graph of GO terms of cellular component enriched in differentially expressed genes in function of the water stress in both SF193 and SF193xSF326 genotypes obtained by the hypergeometric test with the analysis tool of AgriGO.

## 3.6 Supplementary Figure 6


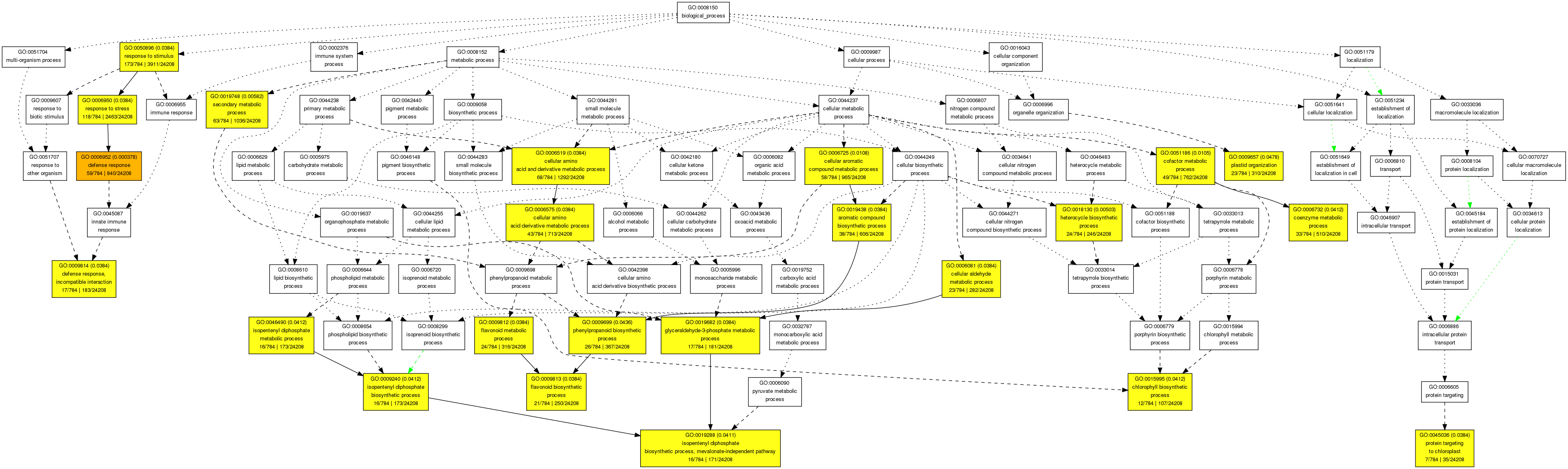
**Supplementary Figure 6.** (Graph_BP_genotype_intersection.png) Hierarchical tree graph of GO terms of biologicat process enriched in differentially expressed genes in function of their genotype in both control and stress conditions obtained by the hypergeometric test with the analysis tool of AgriGO.

## 3.7 Supplementary Figure 7


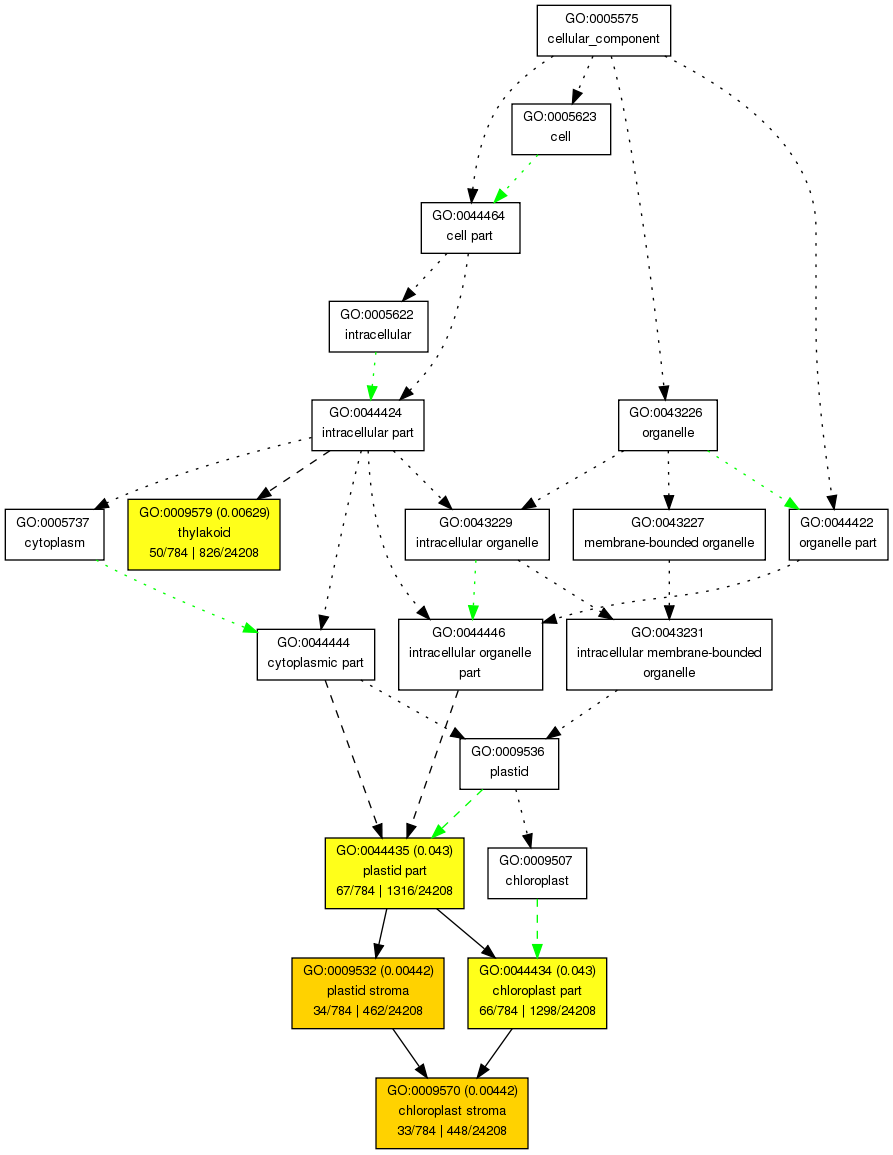
**Supplementary Figure 7.** (Graph_CC_genotype_intersection.png) Hierarchical tree graph of GO terms of cellular component enriched in differentially expressed genes in function of their genotype in both control and stress conditions obtained by the hypergeometric test with the analysis tool of AgriGO.

## 3.8 Supplementary Figure 8


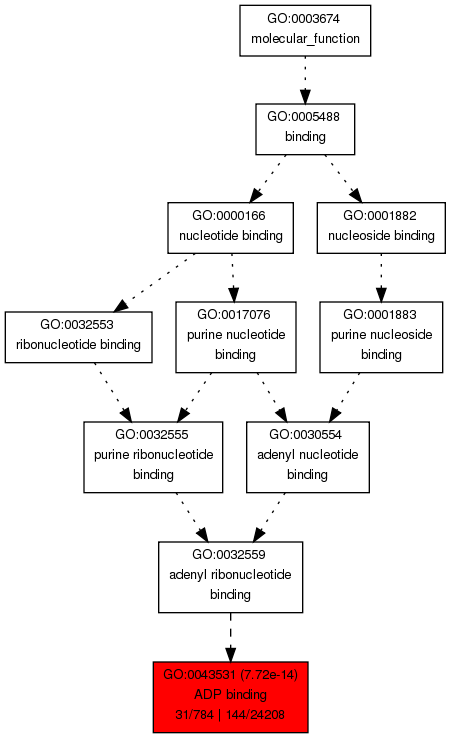
**Supplementary Figure 8.** (Graph_MF_genotype_intersection.png) Hierarchical tree graph of GO terms of molecular fonction enriched in differentially expressed genes in function of their genotype in both control and stress conditions obtained by the hypergeometric test with the analysis tool of AgriGO.
